# {2,2′-[*N*,*N*′-Bis(pyridin-2-ylmeth­yl)propane-1,3-diyldi(nitrilo)]di­acetato}­cobalt(III) hexa­fluoridophosphate aceto­nitrile 0.064-solvate

**DOI:** 10.1107/S1600536813011136

**Published:** 2013-04-30

**Authors:** Craig C. McLauchlan, Daniel S. Kissel, William R. Arnold, Albert W. Herlinger

**Affiliations:** a Department of Chemistry, Illinois State University, Campus Box 4160, Normal, IL 61790-4160, USA; b Department of Chemistry, Loyola University Chicago, Chicago, IL 60626, USA

## Abstract

In the title compound, [Co(C_19_H_22_N_4_O_4_)]PF_6_·0.064CH_3_CN, commonly known as [Co(bppd)]PF_6_·0.064CH_3_CN, where bppd represents the historical ligand name *N*,*N*′-bis(2-pyridylmethyl)-1,3-diaminopropane-*N*,*N*′-diacetate, the Co^III^ atom is coordinated in a distorted octa­hedral geometry with an N_4_O_2_ donor atom set. The acetate O atoms, which exhibit monodentate coordination, are oriented in a *trans* configuration with respect to each other, whereas the pyridyl N atoms are coordinated in a *cis* configuration. The compound crystallizes with two crystallographically unique cations and two anions per asymmetric unit along with a disordered, partially occupied (occupancy = 0.128) aceto­nitrile solvent mol­ecule. Crystals of the title complex were found to be twinned by pseudomerohedry with a 180° rotation around [10-1] and a refined contribution of 90.5 (3)% of the major twin component.

## Related literature

For this and related ligands, see: Lacoste *et al.* (1965 ▶); Caravan *et al.* (1997 ▶); Kanamori *et al.* (2001 ▶); Kissel *et al.* (2013 ▶). For a structure with a derivative of this ligand, see: Sato *et al.* (2012 ▶). For a related Co^III^-*N,N′*-bis­(2-pyridyl­meth­yl)-1,2-di­amino­ethane-*N,N′*-di­acetate complex, [Co(bped)^+^], see: Caravan *et al.* (1997 ▶). For literature on possible applications, see: Caravan *et al.* (1997 ▶); Geraldes (1999 ▶); Jensen (2000 ▶); Heitzmann *et al.* (2009 ▶); Ogden *et al.* (2012 ▶); Sato *et al.* (2012 ▶).
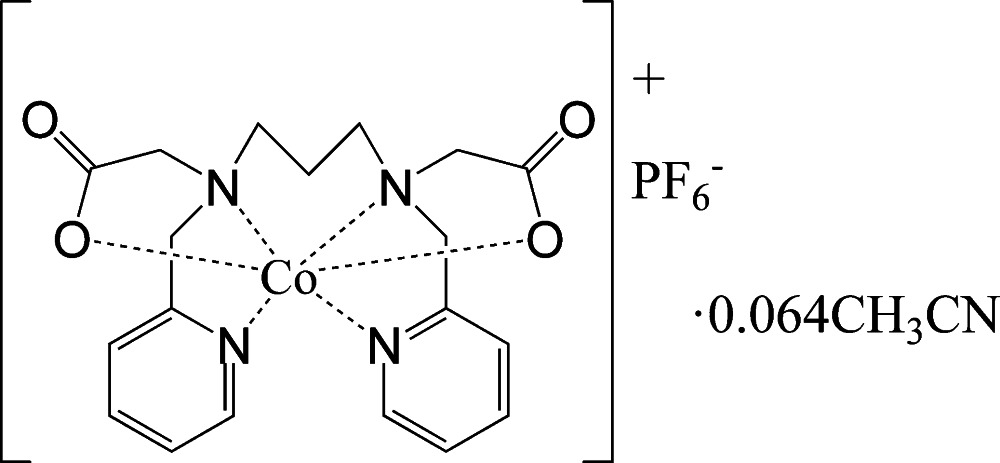


## Experimental

### 

#### Crystal data

[Co(C_19_H_22_N_4_O_4_)]PF_6_·0.064C_2_H_3_N
*M* *_r_* = 576.80
Monoclinic, 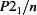

*a* = 21.8891 (7) Å
*b* = 10.2350 (3) Å
*c* = 21.9242 (7) Å
β = 112.802 (2)°
*V* = 4527.9 (2) Å^3^
*Z* = 8
Mo *K*α radiation
μ = 0.91 mm^−1^
*T* = 100 K
0.46 × 0.23 × 0.16 mm

#### Data collection

Bruker APEXII diffractometer equipped with a CCD detector
Absorption correction: multi-scan (*SADABS*; Bruker, 2008 ▶) *T*_min_ = 0.680, *T*_max_ = 0.871
98080 measured reflections
10379 independent reflections
9851 reflections with *I* > 2σ(*I*)
*R*_int_ = 0.028

#### Refinement

*R*[*F* ^2^ > 2σ(*F* ^2^)] = 0.025
*wR*(*F* ^2^) = 0.074
*S* = 1.28
10379 reflections
645 parameters
H-atom parameters constrained
Δρ_max_ = 0.64 e Å^−3^
Δρ_min_ = −0.48 e Å^−3^

### 

Data collection: *APEX2* (Bruker, 2008 ▶); cell refinement: *SAINT* (Bruker, 2008 ▶); data reduction: *SAINT*; program(s) used to solve structure: *SHELXS97* (Sheldrick, 2008 ▶); program(s) used to refine structure: *SHELXL97* (Sheldrick, 2008 ▶); molecular graphics: *ORTEP-3 for Windows* (Farrugia, 2012 ▶); software used to prepare material for publication: *enCIFer* (Allen *et al.*, 2004 ▶).

## Supplementary Material

Crystal structure: contains datablock(s) global, I. DOI: 10.1107/S1600536813011136/wm2736sup1.cif

Structure factors: contains datablock(s) I. DOI: 10.1107/S1600536813011136/wm2736Isup2.hkl

Supplementary material file. DOI: 10.1107/S1600536813011136/wm2736Isup3.cdx

Additional supplementary materials:  crystallographic information; 3D view; checkCIF report

## Figures and Tables

**Table 1:** Selected bond lengths (Å)

Co1—O1	1.8828 (11)
Co1—O3	1.8899 (11)
Co1—N1	1.9484 (13)
Co1—N2	1.9625 (12)
Co1—N3	1.9397 (13)
Co1—N4	1.9641 (13)
Co2—O5	1.8875 (10)
Co2—O7	1.8830 (11)
Co2—N5	1.9403 (13)
Co2—N6	1.9654 (12)
Co2—N7	1.9575 (12)
Co2—N8	1.9645 (12)

**Fig. 1.**
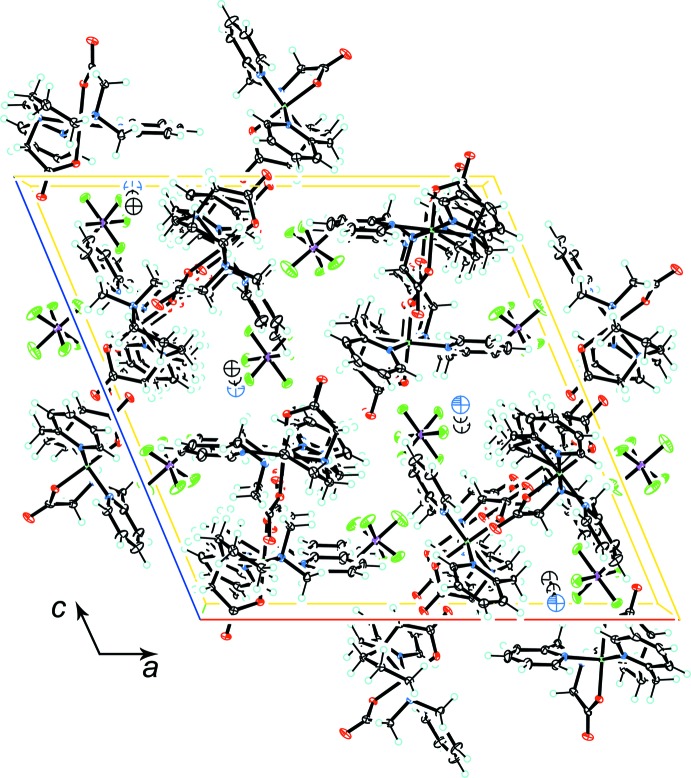
View of the packing of the molecular components of the title compound showing 50% displacement ellipsoids (arbitrary spheres for the H atoms) along [010]. Color code: C: black; H: cyan; Co: aquamarine; F: green; N: blue; O: red; P: magenta.

**Fig. 2.**
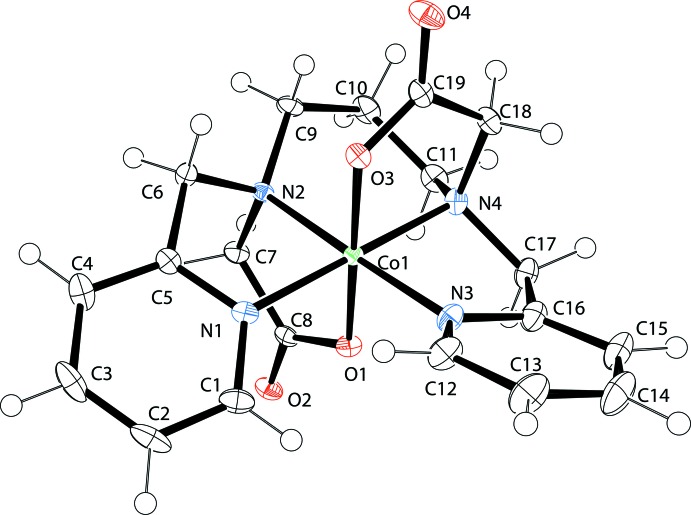
View of the molecular structure of one cation of the title compound showing 50% displacement ellipsoids (arbitrary spheres for the H atoms).

**Fig. 3.**
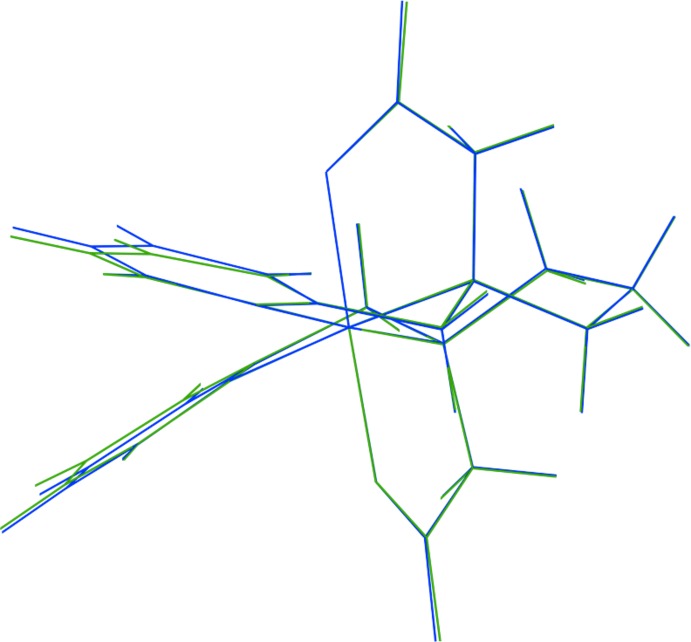
Overlay of the two crystallographically unique cations.

**Fig. 4.**
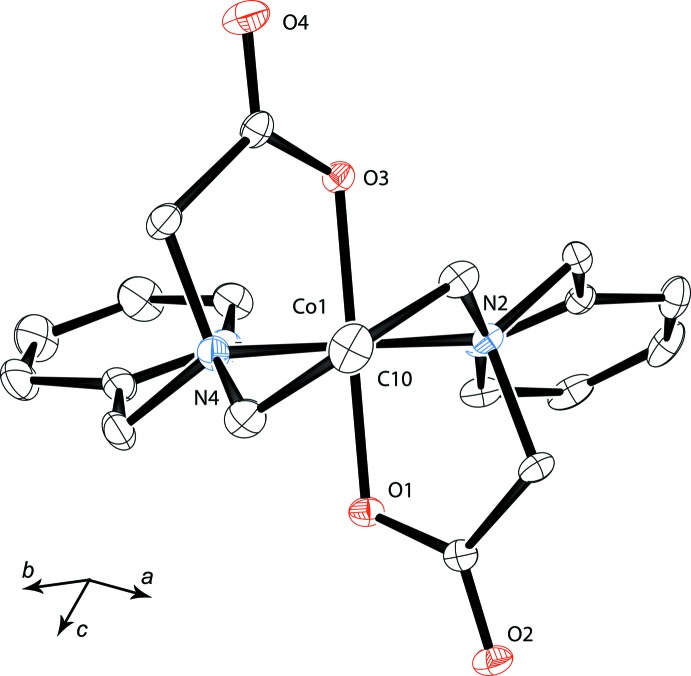
View of the molecular structure of one cation of the title compound down the non-crystallographic 2-fold axis (approximate unit vector -0.290 0.896 0.332). H atoms have been removed for clarity.

## supplementary crystallographic information

### Comment

The title compound, [Co(C_19_H_22_N_4_O_4_)]PF_6_^.^0.064CH_3_CN or [Co(bppd)]PF_6_^.^0.064CH_3_CN, (I), where bppd represents the historical ligand name *N*,*N*'-bis(2-pyridylmethyl)-1,3-diaminopropane-*N*,*N*'-diacetate, was synthesized from *N,N'*-bis(2-pyridylmethyl)-1,3-diaminopropane-*N,N'*-diacetic acid. (H_2_bppd) is the second member of a series of symmetrically substituted polyaminocarboxylic acids with varying diamino backbones. Polyaminocarboxylic acids (Lacoste *et al.*, 1965) and their derivatives are of considerable interest as complexing agents for potential application as magnetic resonance imaging agents when complexed to lanthanides, and solvent extraction reagents for spent nuclear fuel (SNF) reprocessing (Caravan *et al.*, 1997; Geraldes, 1999; Heitzmann *et al.*, 2009). There is very little structural information in the literature about the title ligand and its complexes. There is, however, a structure for a derivative of this ligand and a related Co^III^-*N,N'*-bis(2-pyridylmethyl)-1,2-diaminoethane- *N,N'*-diacetate complex, Co(bped)^+^ (Caravan *et al.*, 1997; Sato *et al.*, 2012).

The H_2_bppd ligand is under investigation for use as a complexing agent for trivalent actinide (*An*(III))-lanthanide (*Ln*(III)) separations. This chemical separation is one of the more difficult challenges in SNF reprocessing because of the very similar physiochemical properties of *An*(III) and *Ln*(III) ions. It has been shown that donor atoms softer than oxygen make this difficult separation more selective, but at the cost of complex stability (Jensen, 2000; Ogden *et al.*, 2012). Polyaminocarboxylic acids, containing softer aromatic nitrogen donors and harder oxygen donors, have been shown to provide good selectivity with adequate stability (Heitzmann *et al.*, 2009). The H_2_bppd ligand features a modified diamine backbone with softer 2-pyridylmethyl substituents to provide selectivity and harder acetate functionalities to improve stability.

The asymmetric unit of compound (I) features two unique, well separated cation/anion pairs with a small amount of CH_3_CN solvate present. The cations and anions are well resolved, but some interactions do appear to be less than the sum of the van der Waals radii; the PF_6_ anion interacts primarily with the methylene unit or pyridine units of the ligand, with distances between 3.0 and 3.4 Å for F···C. A packing diagram of the unit cell is shown in Figure 1.

One of the [Co(bppd)]^+^ cations is shown in Figure 2. The configurations of both cations in the asymmetric unit are very similar, but differ slightly in the pitch of the pyridine rings and the position of the acetate groups; an overlay of the two cations is shown in Figure 3. The deviations are enough to break any higher symmetry. The bond lengths that define the Co^III^ coordination sphere for both cations are listed in Table 1 and a full listing of bond lengths and angles is available in the supplementary materials. The Co^III^ is hexacoordinate with a N_4_O_2_ donor set featuring two neutral tertiary aliphatic amine nitrogen atoms, two neutral aromatic nitrogen atoms, and two anionic acetate oxygen atoms. The complex has a distorted octahedral geometry and has idealized *C*_2_ symmetry, which features a non-crystallographic twofold rotation axis through the cobalt cation and the center carbon of the propylene backbone (Co1···C10 and Co2···C29, respectively), Figure 4. Because of their structural similarity, only one cation will be discussed. The acetate groups, which exhibit monodentate coordination, are oriented in a *trans* configuration with an O1—Co1—O3 angle of 178.47 (5)°. The pyridyl nitrogen atoms are coordinated *cis* with respect to each other defining a N3—Co1—N1 angle of 98.52 (6)°. The bite angle of the diamine backbone is slightly opened to a N2—Co1—N4 angle of 95.91 (5)°. The angles defined by the aliphatic amine nitrogen and pyridyl ring nitrogen, N1—Co1—N2 and N3—Co1—N4, are slightly compressed to 82.36 (5)° and 83.28 (6)°, respectively. The structure of the Co(bppd)^+^ cation is similar, then, to that reported by Caravan *et al.* (1997) for Co(bped)^+^, but with a somewhat less distorted octahedral coordination geometry in the present case.

### Experimental

The H_2_bppd ligand was prepared by a three step synthetic procedure (Kissel *et al.*, 2013) using simple starting materials, which produced higher yields than previously reported methods (Kanamori *et al.*, 2001). The title compound was prepared by slowly adding an equivalent amount of CoCl_2_^.^6H_2_O to a methanolic solution of H_2_bppd^.^3HCl that was neutralized with sodium acetate to the dianion. The mixture was aerated in the presence of activated charcoal while stirring overnight at room temperature. The dark cherry-red mixture was filtered and sodium hexafluoridophosphate added to the filtrate with stirring. A red solid precipitated out of solution upon standing at room temperature for 24 h. The isolated solid was collected by suction filtration and washed with cold methanol; yield: 110 mg (0.2 mmol), 40%. Cherry-red crystals suitable for X-ray diffraction studies were generated by slow evaporation from acetonitrile. All analyses were conducted on the bulk material prior to recrystallization. Analysis on the bulk complex showed the material is isolated as a monohydrate. The water of hydration is apparently lost upon recrystallization from acetonitrile. Anal. obs. (calc.): C, 38.75 (38.53); H, 3.51 (4.08); N 9.24 (9.46). Mag. Susc. µ_eff_ = 0.0 BM. IR (ν(cm^-1^), KBr): 3030 (m, C—H aryl str), 2947 (m, C—H alkyl str), 1660 (vs, b, COO^-^ str), 1610 (s, C═N str), 1471 (m, CH_2_), 1448 (m, C═C str), 1361 (s, COO^-^ str), 1342 (m, C═N str).

### Refinement

The structure of the title complex can be solved and refined in *P*2_1_/*n* routinely with *R*_1_ of *ca* 0.11. Analysis of the data confirms that the crystals are likely a case of twinning by pseudomerohedry with a 180° rotation around [101] in this setting. With inclusion of the twin law, the batch scale factor refines to 0.905 (3) and *R*_1_ values go below 0.03. Although quite satisfactory, residual electron density approximating an acetonitrile, the solvent of cyrstallization, is clearly present and can be further modelled. The occupancy of the CH_3_CN molecule was allowed to vary and refined to a non-chemically meaningful 11%, or 5.5% per cation/anion pair. Upon inspection, the refined bond lengths within the solvent were unreasonable, though, notably the C—C distance, and the molecule was then re-refined with isotropic displacement parameters and H atoms removed resulting in a slightly higher occupancy of 6.4 (5)% per cation/anion pair. As a comparison, another crystal isolated using the same crystallization method refined to an occupancy of 4.5% per cation/anion pair. Efforts to further model both the disorder and the partial occupancy were unfruitful and the overall effort involved does little to improve on the intial *R*_1_ value with no CH_3_CN modelled at all, and makes no significant difference to the cation and anion models of (I).

All H atoms were geometrically placed (C—H = 0.93–0.97 Å) and refined as riding with the exception of the H atoms on the disordered, partially occupied CH_3_CN, which were not modelled, but included in the overall formulation.

### Crystal data

**Table:**

[Co(C_19_H_22_N_4_O_4_)]PF_6_·0.064C_2_H_3_N	*F*(000) = 2346
*M**_r_* = 576.80	*D*_x_ = 1.692 Mg m^−^^3^
Monoclinic, *P*2_1_/*n*	Mo *K*α radiation, λ = 0.71073 Å
*a* = 21.8891 (7) Å	Cell parameters from 9929 reflections
*b* = 10.2350 (3) Å	θ = 2.2–31.9°
*c* = 21.9242 (7) Å	µ = 0.91 mm^−^^1^
β = 112.802 (2)°	*T* = 100 K
*V* = 4527.9 (2) Å^3^	Cut block, translucent dark red-orange
*Z* = 8	0.46 × 0.23 × 0.16 mm

### Data collection

**Table:**

Bruker APEXII diffractometer equipped with a CCD detector	10379 independent reflections
Radiation source: fine-focus sealed tube	9851 reflections with *I* > 2σ(*I*)
Graphite monochromator	*R*_int_ = 0.028
Detector resolution: 8.3333 pixels mm^-1^	θ_max_ = 27.5°, θ_min_ = 1.0°
φ and ω scans	*h* = −28→28
Absorption correction: multi-scan (*SADABS*; Bruker, 2008)	*k* = −13→12
*T*_min_ = 0.680, *T*_max_ = 0.871	*l* = −28→27
98080 measured reflections	

### Refinement

**Table:**

Refinement on *F*^2^	Primary atom site location: structure-invariant direct methods
Least-squares matrix: full	Secondary atom site location: difference Fourier map
*R*[*F*^2^ > 2σ(*F*^2^)] = 0.025	Hydrogen site location: inferred from neighbouring sites
*wR*(*F*^2^) = 0.074	H-atom parameters constrained
*S* = 1.28	*w* = 1/[σ^2^(*F*_o_^2^) + (0.0454*P*)^2^] where *P* = (*F*_o_^2^ + 2*F*_c_^2^)/3
10379 reflections	(Δ/σ)_max_ = 0.001
645 parameters	Δρ_max_ = 0.64 e Å^−^^3^
0 restraints	Δρ_min_ = −0.48 e Å^−^^3^

### Special details

**Table:**

Geometry. All e.s.d.'s (except the e.s.d. in the dihedral angle between two l.s. planes) are estimated using the full covariance matrix. The cell e.s.d.'s are taken into account individually in the estimation of e.s.d.'s in distances, angles and torsion angles; correlations between e.s.d.'s in cell parameters are only used when they are defined by crystal symmetry. An approximate (isotropic) treatment of cell e.s.d.'s is used for estimating e.s.d.'s involving l.s. planes.
Refinement. Refinement of *F*^2^ against ALL reflections. The weighted *R*-factor *wR* and goodness of fit *S* are based on *F*^2^, conventional *R*-factors *R* are based on *F*, with *F* set to zero for negative *F*^2^. The threshold expression of *F*^2^ > σ(*F*^2^) is used only for calculating *R*-factors(gt) *etc*. and is not relevant to the choice of reflections for refinement. *R*-factors based on *F*^2^ are statistically about twice as large as those based on *F*, and *R*- factors based on ALL data will be even larger.

### Fractional atomic coordinates and isotropic or equivalent isotropic displacement parameters (Å^2^)

**Table:**

	*x*	*y*	*z*	*U*_iso_*/*U*_eq_	Occ. (<1)
Co1	0.680379 (10)	0.290022 (18)	0.624729 (10)	0.00956 (5)	
O1	0.63681 (5)	0.32269 (11)	0.53348 (5)	0.0132 (2)	
O2	0.53601 (5)	0.35871 (10)	0.45664 (5)	0.0158 (2)	
O3	0.72325 (5)	0.26154 (11)	0.71680 (5)	0.0127 (2)	
O4	0.78066 (6)	0.36604 (11)	0.81082 (5)	0.0202 (2)	
N1	0.63416 (6)	0.12289 (12)	0.60754 (6)	0.0120 (2)	
N2	0.60034 (6)	0.34784 (12)	0.63686 (6)	0.0105 (2)	
N3	0.76133 (6)	0.23423 (14)	0.61607 (7)	0.0152 (3)	
N4	0.72306 (6)	0.46240 (12)	0.63882 (6)	0.0135 (2)	
C1	0.63753 (8)	0.02776 (15)	0.56666 (8)	0.0175 (3)	
H1A	0.6680	0.0363	0.5456	0.021*	
C2	0.59791 (9)	−0.08206 (16)	0.55439 (8)	0.0223 (4)	
H2A	0.6012	−0.1482	0.5254	0.027*	
C3	0.55346 (8)	−0.09469 (16)	0.58478 (9)	0.0240 (4)	
H3A	0.5271	−0.1711	0.5783	0.029*	
C4	0.54785 (8)	0.00546 (16)	0.62489 (9)	0.0200 (3)	
H4A	0.5169	−0.0004	0.6455	0.024*	
C5	0.58810 (7)	0.11385 (14)	0.63437 (7)	0.0129 (3)	
C6	0.58279 (7)	0.23440 (15)	0.67041 (7)	0.0128 (3)	
H6A	0.6137	0.2296	0.7174	0.015*	
H6B	0.5371	0.2443	0.6686	0.015*	
C7	0.54826 (7)	0.37022 (15)	0.56912 (7)	0.0132 (3)	
H7A	0.5314	0.4607	0.5663	0.016*	
H7B	0.5107	0.3100	0.5620	0.016*	
C8	0.57420 (7)	0.34900 (13)	0.51470 (7)	0.0119 (3)	
C9	0.60885 (7)	0.46390 (14)	0.68086 (7)	0.0138 (3)	
H9A	0.6416	0.4416	0.7255	0.017*	
H9B	0.5661	0.4814	0.6850	0.017*	
C10	0.63131 (8)	0.58850 (15)	0.65802 (8)	0.0184 (3)	
H10A	0.6557	0.6421	0.6976	0.022*	
H10B	0.5913	0.6383	0.6306	0.022*	
C11	0.67509 (8)	0.57374 (15)	0.61848 (8)	0.0161 (3)	
H11A	0.6461	0.5627	0.5712	0.019*	
H11B	0.7003	0.6558	0.6225	0.019*	
C12	0.78860 (8)	0.11362 (17)	0.62926 (8)	0.0201 (3)	
H12A	0.7654	0.0452	0.6404	0.024*	
C13	0.84984 (9)	0.08817 (19)	0.62679 (10)	0.0279 (4)	
H13A	0.8678	0.0024	0.6347	0.033*	
C14	0.88440 (10)	0.1886 (2)	0.61265 (10)	0.0307 (4)	
H14A	0.9259	0.1722	0.6098	0.037*	
C15	0.85800 (9)	0.31391 (19)	0.60268 (9)	0.0245 (4)	
H15A	0.8821	0.3851	0.5952	0.029*	
C16	0.79558 (8)	0.33316 (17)	0.60381 (8)	0.0178 (3)	
C17	0.76100 (8)	0.46245 (16)	0.59474 (8)	0.0177 (3)	
H17A	0.7937	0.5346	0.6070	0.021*	
H17B	0.7305	0.4741	0.5480	0.021*	
C18	0.76896 (7)	0.47169 (15)	0.71010 (7)	0.0154 (3)	
H18A	0.7620	0.5562	0.7284	0.019*	
H18B	0.8154	0.4689	0.7135	0.019*	
C19	0.75738 (7)	0.36034 (14)	0.75052 (8)	0.0137 (3)	
Co2	0.127881 (10)	0.206242 (17)	0.666003 (10)	0.00865 (5)	
O5	0.21806 (5)	0.23182 (10)	0.72040 (5)	0.0121 (2)	
O6	0.30655 (6)	0.12785 (11)	0.79108 (6)	0.0230 (3)	
O7	0.03857 (5)	0.17621 (10)	0.61087 (5)	0.0131 (2)	
O8	−0.02655 (5)	0.14861 (11)	0.50480 (6)	0.0179 (2)	
N5	0.10676 (6)	0.26211 (13)	0.74014 (6)	0.0140 (3)	
N6	0.13431 (6)	0.03316 (12)	0.70686 (6)	0.0119 (2)	
N7	0.11816 (6)	0.37471 (12)	0.62093 (6)	0.0104 (2)	
N8	0.15295 (6)	0.14819 (12)	0.59347 (6)	0.0099 (2)	
C20	0.11881 (8)	0.38126 (16)	0.76838 (8)	0.0180 (3)	
H20A	0.1335	0.4493	0.7479	0.022*	
C21	0.11032 (10)	0.40688 (18)	0.82670 (9)	0.0265 (4)	
H21A	0.1180	0.4920	0.8454	0.032*	
C22	0.09057 (12)	0.30666 (18)	0.85706 (10)	0.0305 (4)	
H22A	0.0847	0.3223	0.8972	0.037*	
C23	0.07924 (10)	0.18297 (18)	0.82882 (10)	0.0262 (4)	
H23A	0.0658	0.1130	0.8492	0.031*	
C24	0.08809 (8)	0.16379 (16)	0.76996 (8)	0.0179 (3)	
C25	0.08214 (8)	0.03443 (15)	0.73538 (8)	0.0168 (3)	
H25A	0.0893	−0.0384	0.7671	0.020*	
H25B	0.0375	0.0250	0.6998	0.020*	
C26	0.20196 (8)	0.02161 (15)	0.76059 (7)	0.0153 (3)	
H26A	0.2220	−0.0626	0.7559	0.018*	
H26B	0.1981	0.0218	0.8041	0.018*	
C27	0.24717 (8)	0.13318 (14)	0.75825 (7)	0.0144 (3)	
C28	0.11960 (7)	−0.07812 (14)	0.65890 (7)	0.0138 (3)	
H28A	0.0746	−0.0659	0.6245	0.017*	
H28B	0.1193	−0.1602	0.6827	0.017*	
C29	0.16849 (8)	−0.09321 (15)	0.62498 (8)	0.0150 (3)	
H29A	0.1460	−0.1422	0.5833	0.018*	
H29B	0.2059	−0.1478	0.6538	0.018*	
C30	0.19734 (7)	0.03175 (14)	0.60843 (7)	0.0127 (3)	
H30A	0.2390	0.0532	0.6462	0.015*	
H30B	0.2088	0.0142	0.5697	0.015*	
C31	0.07496 (7)	0.47038 (14)	0.61800 (7)	0.0126 (3)	
H31A	0.0491	0.4633	0.6440	0.015*	
C32	0.06728 (7)	0.57872 (15)	0.57810 (7)	0.0143 (3)	
H32A	0.0366	0.6454	0.5769	0.017*	
C33	0.10461 (8)	0.58906 (15)	0.54001 (8)	0.0165 (3)	
H33A	0.1008	0.6639	0.5132	0.020*	
C34	0.14785 (8)	0.48846 (15)	0.54148 (8)	0.0173 (3)	
H34A	0.1737	0.4931	0.5154	0.021*	
C35	0.15248 (7)	0.38192 (14)	0.58144 (7)	0.0116 (3)	
C36	0.19088 (7)	0.26107 (15)	0.58220 (7)	0.0123 (3)	
H36A	0.2355	0.2658	0.6180	0.015*	
H36B	0.1960	0.2508	0.5395	0.015*	
C37	0.08966 (7)	0.12739 (15)	0.53421 (7)	0.0128 (3)	
H37A	0.0883	0.0364	0.5185	0.015*	
H37B	0.0889	0.1864	0.4982	0.015*	
C38	0.02848 (7)	0.15290 (14)	0.54956 (8)	0.0128 (3)	
P1	0.87051 (2)	0.20853 (4)	0.09170 (2)	0.01435 (9)	
F1	0.88717 (5)	0.15364 (11)	0.03144 (5)	0.0253 (2)	
F2	0.92037 (5)	0.32889 (10)	0.09903 (5)	0.0214 (2)	
F3	0.93125 (5)	0.12522 (10)	0.14210 (5)	0.0245 (2)	
F4	0.85452 (5)	0.26333 (12)	0.15215 (6)	0.0293 (2)	
F5	0.81102 (5)	0.29279 (11)	0.04093 (6)	0.0320 (3)	
F6	0.82114 (5)	0.08857 (11)	0.08447 (6)	0.0309 (2)	
P2	0.56387 (2)	0.20817 (4)	0.84196 (2)	0.01808 (9)	
F7	0.56344 (7)	0.09114 (13)	0.89065 (6)	0.0421 (3)	
F8	0.56504 (7)	0.32505 (11)	0.79448 (6)	0.0385 (3)	
F9	0.63582 (6)	0.24718 (17)	0.89367 (8)	0.0553 (4)	
F10	0.59708 (7)	0.11130 (12)	0.80632 (6)	0.0420 (3)	
F11	0.53192 (7)	0.30432 (13)	0.87873 (6)	0.0408 (3)	
F12	0.49280 (7)	0.16633 (14)	0.79232 (8)	0.0552 (4)	
C1S	0.7245 (10)	0.1996 (18)	0.4578 (10)	0.041 (5)*	0.128 (5)
C2S	0.7208 (12)	0.319 (2)	0.4357 (12)	0.060 (6)*	0.128 (5)
N1S	0.7325 (11)	0.094 (2)	0.4837 (11)	0.069 (6)*	0.128 (5)

### Atomic displacement parameters (Å^2^)

**Table:**

	*U*^11^	*U*^22^	*U*^33^	*U*^12^	*U*^13^	*U*^23^
Co1	0.00947 (10)	0.00884 (10)	0.01061 (10)	0.00142 (7)	0.00414 (8)	0.00024 (7)
O1	0.0143 (5)	0.0130 (5)	0.0118 (5)	0.0028 (4)	0.0047 (4)	0.0005 (4)
O2	0.0184 (5)	0.0142 (5)	0.0110 (5)	−0.0002 (4)	0.0015 (4)	−0.0007 (4)
O3	0.0124 (5)	0.0112 (5)	0.0132 (5)	0.0005 (4)	0.0034 (4)	0.0005 (4)
O4	0.0236 (6)	0.0165 (6)	0.0138 (5)	−0.0006 (5)	−0.0001 (5)	0.0005 (4)
N1	0.0135 (6)	0.0101 (6)	0.0105 (6)	0.0026 (5)	0.0028 (5)	0.0005 (4)
N2	0.0104 (6)	0.0097 (6)	0.0105 (6)	0.0011 (4)	0.0030 (5)	−0.0015 (4)
N3	0.0139 (6)	0.0168 (6)	0.0165 (7)	0.0033 (5)	0.0076 (5)	0.0027 (5)
N4	0.0122 (6)	0.0120 (6)	0.0158 (6)	0.0009 (5)	0.0050 (5)	0.0027 (5)
C1	0.0214 (8)	0.0148 (7)	0.0137 (7)	0.0055 (6)	0.0040 (6)	−0.0010 (6)
C2	0.0243 (8)	0.0134 (7)	0.0190 (8)	0.0043 (6)	−0.0030 (7)	−0.0066 (6)
C3	0.0158 (8)	0.0106 (7)	0.0346 (10)	−0.0009 (6)	−0.0024 (7)	−0.0018 (7)
C4	0.0138 (7)	0.0142 (8)	0.0291 (9)	−0.0003 (6)	0.0052 (6)	0.0024 (6)
C5	0.0111 (7)	0.0119 (7)	0.0134 (7)	0.0018 (5)	0.0023 (5)	0.0012 (5)
C6	0.0126 (7)	0.0126 (7)	0.0143 (7)	−0.0001 (5)	0.0065 (6)	0.0007 (6)
C7	0.0110 (7)	0.0146 (7)	0.0111 (7)	0.0028 (5)	0.0011 (5)	−0.0002 (5)
C8	0.0156 (7)	0.0058 (6)	0.0129 (7)	−0.0006 (5)	0.0041 (6)	−0.0010 (5)
C9	0.0149 (7)	0.0113 (7)	0.0141 (7)	0.0021 (5)	0.0043 (6)	−0.0041 (5)
C10	0.0185 (8)	0.0101 (7)	0.0250 (8)	0.0019 (6)	0.0068 (6)	−0.0031 (6)
C11	0.0176 (7)	0.0097 (7)	0.0185 (8)	0.0019 (6)	0.0044 (6)	0.0025 (5)
C12	0.0199 (8)	0.0198 (8)	0.0224 (8)	0.0078 (6)	0.0102 (7)	0.0048 (6)
C13	0.0245 (9)	0.0287 (10)	0.0348 (10)	0.0135 (7)	0.0165 (8)	0.0071 (8)
C14	0.0183 (9)	0.0432 (11)	0.0364 (11)	0.0094 (8)	0.0168 (8)	0.0067 (9)
C15	0.0192 (8)	0.0332 (10)	0.0260 (9)	0.0016 (7)	0.0143 (7)	0.0063 (7)
C16	0.0159 (7)	0.0216 (8)	0.0171 (8)	0.0004 (6)	0.0078 (6)	0.0036 (6)
C17	0.0179 (7)	0.0178 (8)	0.0204 (8)	−0.0022 (6)	0.0106 (6)	0.0038 (6)
C18	0.0128 (7)	0.0146 (7)	0.0153 (7)	−0.0015 (6)	0.0014 (6)	−0.0002 (6)
C19	0.0098 (6)	0.0120 (7)	0.0169 (7)	0.0021 (5)	0.0025 (6)	0.0001 (5)
Co2	0.00870 (10)	0.00702 (10)	0.01076 (10)	0.00047 (6)	0.00437 (7)	0.00104 (6)
O5	0.0113 (5)	0.0097 (5)	0.0129 (5)	0.0006 (4)	0.0019 (4)	−0.0003 (4)
O6	0.0172 (6)	0.0159 (6)	0.0240 (6)	0.0014 (4)	−0.0050 (5)	−0.0001 (5)
O7	0.0097 (5)	0.0122 (5)	0.0175 (5)	−0.0004 (4)	0.0052 (4)	0.0016 (4)
O8	0.0115 (5)	0.0128 (5)	0.0225 (6)	0.0009 (4)	−0.0011 (4)	0.0004 (4)
N5	0.0172 (6)	0.0109 (6)	0.0159 (6)	0.0020 (5)	0.0087 (5)	0.0016 (5)
N6	0.0138 (6)	0.0087 (6)	0.0141 (6)	0.0004 (5)	0.0063 (5)	0.0013 (5)
N7	0.0082 (5)	0.0096 (6)	0.0124 (6)	−0.0003 (4)	0.0030 (5)	0.0014 (4)
N8	0.0088 (5)	0.0090 (6)	0.0116 (6)	0.0006 (4)	0.0038 (5)	0.0001 (4)
C20	0.0247 (8)	0.0125 (7)	0.0202 (8)	0.0029 (6)	0.0124 (7)	0.0018 (6)
C21	0.0446 (11)	0.0164 (8)	0.0269 (9)	0.0030 (7)	0.0230 (8)	−0.0013 (7)
C22	0.0506 (13)	0.0233 (10)	0.0290 (10)	0.0032 (8)	0.0278 (10)	−0.0013 (7)
C23	0.0420 (11)	0.0176 (8)	0.0313 (10)	0.0024 (7)	0.0277 (9)	0.0047 (7)
C24	0.0216 (8)	0.0140 (7)	0.0234 (8)	0.0026 (6)	0.0146 (7)	0.0034 (6)
C25	0.0223 (8)	0.0115 (7)	0.0221 (8)	0.0003 (6)	0.0145 (7)	0.0033 (6)
C26	0.0191 (7)	0.0105 (7)	0.0136 (7)	0.0021 (6)	0.0033 (6)	0.0021 (5)
C27	0.0177 (7)	0.0097 (7)	0.0126 (7)	0.0012 (5)	0.0026 (6)	−0.0025 (5)
C28	0.0158 (7)	0.0080 (6)	0.0166 (7)	−0.0011 (5)	0.0051 (6)	−0.0011 (5)
C29	0.0182 (7)	0.0092 (7)	0.0174 (7)	0.0018 (5)	0.0068 (6)	−0.0006 (5)
C30	0.0114 (7)	0.0102 (7)	0.0163 (7)	0.0032 (5)	0.0051 (6)	−0.0008 (5)
C31	0.0105 (6)	0.0117 (7)	0.0147 (7)	0.0004 (5)	0.0039 (5)	−0.0006 (5)
C32	0.0131 (7)	0.0112 (7)	0.0164 (7)	0.0023 (5)	0.0032 (6)	−0.0001 (5)
C33	0.0213 (8)	0.0116 (7)	0.0162 (7)	0.0005 (6)	0.0069 (6)	0.0034 (6)
C34	0.0205 (8)	0.0149 (7)	0.0205 (8)	0.0013 (6)	0.0122 (6)	0.0034 (6)
C35	0.0100 (6)	0.0114 (7)	0.0134 (7)	−0.0006 (5)	0.0045 (5)	−0.0012 (5)
C36	0.0113 (7)	0.0108 (7)	0.0166 (7)	−0.0002 (5)	0.0074 (6)	0.0019 (5)
C37	0.0114 (7)	0.0131 (7)	0.0116 (7)	−0.0005 (5)	0.0020 (5)	−0.0003 (5)
C38	0.0123 (7)	0.0055 (6)	0.0192 (7)	−0.0001 (5)	0.0045 (6)	0.0020 (5)
P1	0.01266 (19)	0.0157 (2)	0.0164 (2)	−0.00268 (14)	0.00746 (16)	−0.00115 (14)
F1	0.0283 (5)	0.0311 (6)	0.0205 (5)	−0.0050 (4)	0.0138 (4)	−0.0074 (4)
F2	0.0183 (5)	0.0168 (5)	0.0318 (5)	−0.0049 (4)	0.0125 (4)	−0.0017 (4)
F3	0.0234 (5)	0.0265 (5)	0.0243 (5)	0.0032 (4)	0.0100 (4)	0.0101 (4)
F4	0.0286 (6)	0.0389 (6)	0.0290 (6)	−0.0046 (5)	0.0206 (5)	−0.0105 (5)
F5	0.0156 (5)	0.0417 (7)	0.0368 (7)	0.0072 (4)	0.0082 (5)	0.0138 (5)
F6	0.0281 (6)	0.0280 (6)	0.0400 (6)	−0.0157 (5)	0.0171 (5)	−0.0052 (5)
P2	0.0187 (2)	0.0220 (2)	0.0166 (2)	0.00141 (15)	0.01012 (17)	0.00088 (15)
F7	0.0496 (8)	0.0452 (7)	0.0451 (7)	0.0207 (6)	0.0332 (6)	0.0262 (6)
F8	0.0730 (9)	0.0196 (5)	0.0417 (7)	0.0028 (6)	0.0428 (7)	0.0037 (5)
F9	0.0223 (6)	0.0543 (8)	0.0703 (10)	0.0002 (6)	−0.0028 (6)	−0.0132 (8)
F10	0.0770 (9)	0.0252 (6)	0.0433 (7)	0.0162 (6)	0.0447 (7)	0.0068 (5)
F11	0.0529 (8)	0.0519 (8)	0.0260 (6)	0.0294 (6)	0.0246 (6)	0.0077 (5)
F12	0.0358 (7)	0.0416 (8)	0.0599 (9)	−0.0092 (6)	−0.0123 (7)	0.0032 (7)

### Geometric parameters (Å, º)

**Table:**

Co1—O1	1.8828 (11)	O6—C27	1.219 (2)
Co1—O3	1.8899 (11)	O7—C38	1.2971 (19)
Co1—N1	1.9484 (13)	O8—C38	1.2246 (18)
Co1—N2	1.9625 (12)	N5—C20	1.347 (2)
Co1—N3	1.9397 (13)	N5—C24	1.347 (2)
Co1—N4	1.9641 (13)	N6—C26	1.4974 (19)
O1—C8	1.2973 (18)	N6—C28	1.4980 (19)
O2—C8	1.2265 (18)	N6—C25	1.4995 (19)
O3—C19	1.3035 (19)	N7—C31	1.3454 (18)
O4—C19	1.2201 (19)	N7—C35	1.3503 (19)
N1—C1	1.3446 (19)	N8—C30	1.4915 (18)
N1—C5	1.353 (2)	N8—C36	1.4981 (18)
N2—C9	1.4957 (18)	N8—C37	1.5026 (18)
N2—C7	1.4985 (18)	C20—C21	1.386 (2)
N2—C6	1.5019 (19)	C20—H20A	0.9500
N3—C16	1.348 (2)	C21—C22	1.380 (3)
N3—C12	1.353 (2)	C21—H21A	0.9500
N4—C11	1.4956 (19)	C22—C23	1.389 (3)
N4—C17	1.4986 (19)	C22—H22A	0.9500
N4—C18	1.4979 (19)	C23—C24	1.391 (2)
C1—C2	1.381 (2)	C23—H23A	0.9500
C1—H1A	0.9500	C24—C25	1.506 (2)
C2—C3	1.382 (3)	C25—H25A	0.9900
C2—H2A	0.9500	C25—H25B	0.9900
C3—C4	1.387 (2)	C26—C27	1.525 (2)
C3—H3A	0.9500	C26—H26A	0.9900
C4—C5	1.381 (2)	C26—H26B	0.9900
C4—H4A	0.9500	C28—C29	1.529 (2)
C5—C6	1.494 (2)	C28—H28A	0.9900
C6—H6A	0.9900	C28—H28B	0.9900
C6—H6B	0.9900	C29—C30	1.531 (2)
C7—C8	1.522 (2)	C29—H29A	0.9900
C7—H7A	0.9900	C29—H29B	0.9900
C7—H7B	0.9900	C30—H30A	0.9900
C9—C10	1.520 (2)	C30—H30B	0.9900
C9—H9A	0.9900	C31—C32	1.382 (2)
C9—H9B	0.9900	C31—H31A	0.9500
C10—C11	1.529 (2)	C32—C33	1.380 (2)
C10—H10A	0.9900	C32—H32A	0.9500
C10—H10B	0.9900	C33—C34	1.390 (2)
C11—H11A	0.9900	C33—H33A	0.9500
C11—H11B	0.9900	C34—C35	1.378 (2)
C12—C13	1.387 (2)	C34—H34A	0.9500
C12—H12A	0.9500	C35—C36	1.492 (2)
C13—C14	1.381 (3)	C36—H36A	0.9900
C13—H13A	0.9500	C36—H36B	0.9900
C14—C15	1.388 (3)	C37—C38	1.525 (2)
C14—H14A	0.9500	C37—H37A	0.9900
C15—C16	1.390 (2)	C37—H37B	0.9900
C15—H15A	0.9500	P1—F4	1.5979 (11)
C16—C17	1.499 (2)	P1—F5	1.5985 (11)
C17—H17A	0.9900	P1—F6	1.6024 (11)
C17—H17B	0.9900	P1—F1	1.6023 (10)
C18—C19	1.524 (2)	P1—F3	1.6052 (10)
C18—H18A	0.9900	P1—F2	1.6120 (10)
C18—H18B	0.9900	P2—F12	1.5734 (13)
Co2—O5	1.8875 (10)	P2—F8	1.5924 (12)
Co2—O7	1.8830 (11)	P2—F9	1.5941 (14)
Co2—N5	1.9403 (13)	P2—F11	1.5952 (11)
Co2—N6	1.9654 (12)	P2—F10	1.6007 (12)
Co2—N7	1.9575 (12)	P2—F7	1.6069 (12)
Co2—N8	1.9645 (12)	C1S—N1S	1.20 (3)
O5—C27	1.3054 (18)	C1S—C2S	1.30 (3)
			
O1—Co1—O3	178.47 (5)	C38—O7—Co2	114.38 (9)
O1—Co1—N3	93.10 (5)	C20—N5—C24	119.71 (14)
O3—Co1—N3	87.98 (5)	C20—N5—Co2	125.75 (11)
O1—Co1—N1	86.51 (5)	C24—N5—Co2	113.71 (11)
O3—Co1—N1	94.41 (5)	C26—N6—C28	111.73 (11)
N3—Co1—N1	98.52 (6)	C26—N6—C25	110.69 (12)
O1—Co1—N2	88.81 (5)	C28—N6—C25	107.94 (11)
O3—Co1—N2	90.09 (5)	C26—N6—Co2	107.70 (9)
N3—Co1—N2	177.94 (5)	C28—N6—Co2	114.04 (9)
N1—Co1—N2	82.36 (5)	C25—N6—Co2	104.52 (9)
O1—Co1—N4	91.25 (5)	C31—N7—C35	119.02 (13)
O3—Co1—N4	87.79 (5)	C31—N7—Co2	127.02 (10)
N3—Co1—N4	83.28 (6)	C35—N7—Co2	113.15 (10)
N1—Co1—N4	177.20 (5)	C30—N8—C36	107.28 (11)
N2—Co1—N4	95.91 (5)	C30—N8—C37	112.37 (11)
C8—O1—Co1	114.32 (9)	C36—N8—C37	110.53 (11)
C19—O3—Co1	115.11 (10)	C30—N8—Co2	115.23 (9)
C1—N1—C5	118.88 (13)	C36—N8—Co2	104.29 (9)
C1—N1—Co1	127.31 (11)	C37—N8—Co2	106.83 (9)
C5—N1—Co1	113.23 (10)	N5—C20—C21	121.52 (15)
C9—N2—C7	112.00 (11)	N5—C20—H20A	119.2
C9—N2—C6	106.84 (11)	C21—C20—H20A	119.2
C7—N2—C6	111.28 (11)	C22—C21—C20	118.90 (17)
C9—N2—Co1	115.61 (9)	C22—C21—H21A	120.6
C7—N2—Co1	106.74 (9)	C20—C21—H21A	120.6
C6—N2—Co1	104.15 (9)	C21—C22—C23	119.84 (17)
C16—N3—C12	119.65 (14)	C21—C22—H22A	120.1
C16—N3—Co1	113.47 (11)	C23—C22—H22A	120.1
C12—N3—Co1	126.30 (11)	C22—C23—C24	118.51 (16)
C11—N4—C17	107.94 (12)	C22—C23—H23A	120.7
C11—N4—C18	111.85 (12)	C24—C23—H23A	120.7
C17—N4—C18	110.95 (12)	N5—C24—C23	121.49 (16)
C11—N4—Co1	113.66 (9)	N5—C24—C25	113.32 (13)
C17—N4—Co1	104.33 (9)	C23—C24—C25	125.10 (15)
C18—N4—Co1	107.87 (9)	N6—C25—C24	106.63 (12)
N1—C1—C2	121.78 (15)	N6—C25—H25A	110.4
N1—C1—H1A	119.1	C24—C25—H25A	110.4
C2—C1—H1A	119.1	N6—C25—H25B	110.4
C3—C2—C1	119.25 (15)	C24—C25—H25B	110.4
C3—C2—H2A	120.4	H25A—C25—H25B	108.6
C1—C2—H2A	120.4	N6—C26—C27	111.79 (12)
C2—C3—C4	119.25 (15)	N6—C26—H26A	109.3
C2—C3—H3A	120.4	C27—C26—H26A	109.3
C4—C3—H3A	120.4	N6—C26—H26B	109.3
C5—C4—C3	118.74 (16)	C27—C26—H26B	109.3
C5—C4—H4A	120.6	H26A—C26—H26B	107.9
C3—C4—H4A	120.6	O6—C27—O5	123.80 (14)
N1—C5—C4	121.92 (14)	O6—C27—C26	120.27 (14)
N1—C5—C6	113.41 (13)	O5—C27—C26	115.93 (13)
C4—C5—C6	124.54 (14)	N6—C28—C29	114.68 (12)
C5—C6—N2	107.00 (12)	N6—C28—H28A	108.6
C5—C6—H6A	110.3	C29—C28—H28A	108.6
N2—C6—H6A	110.3	N6—C28—H28B	108.6
C5—C6—H6B	110.3	C29—C28—H28B	108.6
N2—C6—H6B	110.3	H28A—C28—H28B	107.6
H6A—C6—H6B	108.6	C28—C29—C30	117.49 (12)
N2—C7—C8	112.54 (12)	C28—C29—H29A	107.9
N2—C7—H7A	109.1	C30—C29—H29A	107.9
C8—C7—H7A	109.1	C28—C29—H29B	107.9
N2—C7—H7B	109.1	C30—C29—H29B	107.9
C8—C7—H7B	109.1	H29A—C29—H29B	107.2
H7A—C7—H7B	107.8	N8—C30—C29	115.23 (12)
O2—C8—O1	123.87 (14)	N8—C30—H30A	108.5
O2—C8—C7	119.39 (13)	C29—C30—H30A	108.5
O1—C8—C7	116.74 (12)	N8—C30—H30B	108.5
N2—C9—C10	115.42 (12)	C29—C30—H30B	108.5
N2—C9—H9A	108.4	H30A—C30—H30B	107.5
C10—C9—H9A	108.4	N7—C31—C32	121.68 (14)
N2—C9—H9B	108.4	N7—C31—H31A	119.2
C10—C9—H9B	108.4	C32—C31—H31A	119.2
H9A—C9—H9B	107.5	C31—C32—C33	119.29 (14)
C9—C10—C11	117.25 (13)	C31—C32—H32A	120.4
C9—C10—H10A	108.0	C33—C32—H32A	120.4
C11—C10—H10A	108.0	C32—C33—C34	119.12 (14)
C9—C10—H10B	108.0	C32—C33—H33A	120.4
C11—C10—H10B	108.0	C34—C33—H33A	120.4
H10A—C10—H10B	107.2	C35—C34—C33	118.84 (14)
N4—C11—C10	115.36 (13)	C35—C34—H34A	120.6
N4—C11—H11A	108.4	C33—C34—H34A	120.6
C10—C11—H11A	108.4	N7—C35—C34	121.93 (14)
N4—C11—H11B	108.4	N7—C35—C36	113.50 (13)
C10—C11—H11B	108.4	C34—C35—C36	124.43 (13)
H11A—C11—H11B	107.5	C35—C36—N8	107.17 (11)
N3—C12—C13	121.08 (16)	C35—C36—H36A	110.3
N3—C12—H12A	119.5	N8—C36—H36A	110.3
C13—C12—H12A	119.5	C35—C36—H36B	110.3
C14—C13—C12	119.38 (17)	N8—C36—H36B	110.3
C14—C13—H13A	120.3	H36A—C36—H36B	108.5
C12—C13—H13A	120.3	N8—C37—C38	112.29 (12)
C13—C14—C15	119.48 (17)	N8—C37—H37A	109.1
C13—C14—H14A	120.3	C38—C37—H37A	109.1
C15—C14—H14A	120.3	N8—C37—H37B	109.1
C14—C15—C16	118.69 (17)	C38—C37—H37B	109.1
C14—C15—H15A	120.7	H37A—C37—H37B	107.9
C16—C15—H15A	120.7	O8—C38—O7	123.82 (14)
N3—C16—C15	121.58 (16)	O8—C38—C37	119.50 (14)
N3—C16—C17	113.48 (13)	O7—C38—C37	116.67 (12)
C15—C16—C17	124.89 (15)	F4—P1—F5	90.40 (7)
N4—C17—C16	106.96 (12)	F4—P1—F6	89.70 (6)
N4—C17—H17A	110.3	F5—P1—F6	90.47 (6)
C16—C17—H17A	110.3	F4—P1—F1	179.54 (6)
N4—C17—H17B	110.3	F5—P1—F1	89.96 (6)
C16—C17—H17B	110.3	F6—P1—F1	90.57 (6)
H17A—C17—H17B	108.6	F4—P1—F3	90.27 (6)
N4—C18—C19	111.33 (12)	F5—P1—F3	178.85 (6)
N4—C18—H18A	109.4	F6—P1—F3	90.46 (6)
C19—C18—H18A	109.4	F1—P1—F3	89.36 (6)
N4—C18—H18B	109.4	F4—P1—F2	90.37 (6)
C19—C18—H18B	109.4	F5—P1—F2	89.69 (6)
H18A—C18—H18B	108.0	F6—P1—F2	179.82 (6)
O4—C19—O3	123.95 (14)	F1—P1—F2	89.35 (6)
O4—C19—C18	120.04 (13)	F3—P1—F2	89.38 (5)
O3—C19—C18	116.00 (13)	F12—P2—F8	90.83 (8)
O7—Co2—O5	178.36 (5)	F12—P2—F9	178.31 (9)
O7—Co2—N5	93.48 (5)	F8—P2—F9	90.86 (9)
O5—Co2—N5	87.72 (5)	F12—P2—F11	90.45 (9)
O7—Co2—N7	86.39 (5)	F8—P2—F11	89.32 (6)
O5—Co2—N7	94.55 (5)	F9—P2—F11	89.53 (8)
N5—Co2—N7	98.55 (5)	F12—P2—F10	90.47 (9)
O7—Co2—N8	88.69 (5)	F8—P2—F10	91.19 (6)
O5—Co2—N8	90.09 (5)	F9—P2—F10	89.54 (8)
N5—Co2—N8	177.67 (5)	F11—P2—F10	178.94 (8)
N7—Co2—N8	82.39 (5)	F12—P2—F7	89.87 (8)
O7—Co2—N6	91.07 (5)	F8—P2—F7	179.26 (9)
O5—Co2—N6	87.96 (5)	F9—P2—F7	88.44 (9)
N5—Co2—N6	83.23 (5)	F11—P2—F7	90.43 (7)
N7—Co2—N6	176.98 (5)	F10—P2—F7	89.04 (6)
N8—Co2—N6	95.92 (5)	N1S—C1S—C2S	174 (2)
C27—O5—Co2	115.33 (10)		
			
O3—Co1—O1—C8	−53.5 (19)	O7—Co2—O5—C27	−51.5 (17)
N3—Co1—O1—C8	171.78 (10)	N5—Co2—O5—C27	85.46 (11)
N1—Co1—O1—C8	73.42 (10)	N7—Co2—O5—C27	−176.14 (10)
N2—Co1—O1—C8	−9.00 (10)	N8—Co2—O5—C27	−93.77 (10)
N4—Co1—O1—C8	−104.89 (10)	N6—Co2—O5—C27	2.16 (10)
O1—Co1—O3—C19	−47.7 (19)	O5—Co2—O7—C38	−52.4 (17)
N3—Co1—O3—C19	87.11 (11)	N5—Co2—O7—C38	170.64 (10)
N1—Co1—O3—C19	−174.50 (10)	N7—Co2—O7—C38	72.28 (10)
N2—Co1—O3—C19	−92.15 (10)	N8—Co2—O7—C38	−10.18 (10)
N4—Co1—O3—C19	3.76 (10)	N6—Co2—O7—C38	−106.08 (10)
O1—Co1—N1—C1	59.18 (13)	O7—Co2—N5—C20	−117.75 (13)
O3—Co1—N1—C1	−122.05 (13)	O5—Co2—N5—C20	63.37 (13)
N3—Co1—N1—C1	−33.44 (13)	N7—Co2—N5—C20	−30.88 (14)
N2—Co1—N1—C1	148.45 (13)	N8—Co2—N5—C20	82.9 (14)
N4—Co1—N1—C1	96.2 (11)	N6—Co2—N5—C20	151.59 (14)
O1—Co1—N1—C5	−111.91 (10)	O7—Co2—N5—C24	72.84 (12)
O3—Co1—N1—C5	66.86 (10)	O5—Co2—N5—C24	−106.03 (12)
N3—Co1—N1—C5	155.48 (10)	N7—Co2—N5—C24	159.71 (11)
N2—Co1—N1—C5	−22.64 (10)	N8—Co2—N5—C24	−86.5 (14)
N4—Co1—N1—C5	−74.9 (11)	N6—Co2—N5—C24	−17.82 (11)
O1—Co1—N2—C9	−119.10 (10)	O7—Co2—N6—C26	−176.35 (9)
O3—Co1—N2—C9	59.83 (10)	O5—Co2—N6—C26	4.98 (9)
N3—Co1—N2—C9	38.8 (16)	N5—Co2—N6—C26	−82.97 (10)
N1—Co1—N2—C9	154.26 (10)	N7—Co2—N6—C26	150.9 (9)
N4—Co1—N2—C9	−27.96 (10)	N8—Co2—N6—C26	94.86 (9)
O1—Co1—N2—C7	6.21 (9)	O7—Co2—N6—C28	59.05 (10)
O3—Co1—N2—C7	−174.87 (9)	O5—Co2—N6—C28	−119.62 (10)
N3—Co1—N2—C7	164.1 (15)	N5—Co2—N6—C28	152.43 (10)
N1—Co1—N2—C7	−80.43 (9)	N7—Co2—N6—C28	26.3 (10)
N4—Co1—N2—C7	97.34 (9)	N8—Co2—N6—C28	−29.74 (10)
O1—Co1—N2—C6	124.02 (9)	O7—Co2—N6—C25	−58.59 (9)
O3—Co1—N2—C6	−57.05 (9)	O5—Co2—N6—C25	122.74 (9)
N3—Co1—N2—C6	−78.1 (15)	N5—Co2—N6—C25	34.79 (9)
N1—Co1—N2—C6	37.38 (9)	N7—Co2—N6—C25	−91.3 (10)
N4—Co1—N2—C6	−144.85 (9)	N8—Co2—N6—C25	−147.38 (9)
O1—Co1—N3—C16	72.52 (12)	O7—Co2—N7—C31	59.07 (12)
O3—Co1—N3—C16	−106.39 (12)	O5—Co2—N7—C31	−122.29 (12)
N1—Co1—N3—C16	159.45 (11)	N5—Co2—N7—C31	−33.91 (13)
N2—Co1—N3—C16	−85.4 (15)	N8—Co2—N7—C31	148.23 (13)
N4—Co1—N3—C16	−18.37 (11)	N6—Co2—N7—C31	91.9 (10)
O1—Co1—N3—C12	−116.33 (14)	O7—Co2—N7—C35	−110.35 (10)
O3—Co1—N3—C12	64.76 (14)	O5—Co2—N7—C35	68.29 (10)
N1—Co1—N3—C12	−29.40 (15)	N5—Co2—N7—C35	156.66 (10)
N2—Co1—N3—C12	85.8 (16)	N8—Co2—N7—C35	−21.19 (10)
N4—Co1—N3—C12	152.78 (15)	N6—Co2—N7—C35	−77.6 (10)
O1—Co1—N4—C11	59.21 (10)	O7—Co2—N8—C30	−119.56 (10)
O3—Co1—N4—C11	−119.60 (10)	O5—Co2—N8—C30	59.34 (10)
N3—Co1—N4—C11	152.18 (11)	N5—Co2—N8—C30	39.8 (14)
N1—Co1—N4—C11	22.2 (12)	N7—Co2—N8—C30	153.91 (10)
N2—Co1—N4—C11	−29.73 (11)	N6—Co2—N8—C30	−28.62 (10)
O1—Co1—N4—C17	−58.12 (9)	O7—Co2—N8—C36	123.12 (9)
O3—Co1—N4—C17	123.08 (9)	O5—Co2—N8—C36	−57.98 (9)
N3—Co1—N4—C17	34.86 (9)	N5—Co2—N8—C36	−77.5 (14)
N1—Co1—N4—C17	−95.1 (11)	N7—Co2—N8—C36	36.59 (9)
N2—Co1—N4—C17	−147.05 (9)	N6—Co2—N8—C36	−145.94 (9)
O1—Co1—N4—C18	−176.16 (9)	O7—Co2—N8—C37	6.05 (9)
O3—Co1—N4—C18	5.04 (9)	O5—Co2—N8—C37	−175.06 (9)
N3—Co1—N4—C18	−83.19 (10)	N5—Co2—N8—C37	165.4 (13)
N1—Co1—N4—C18	146.9 (11)	N7—Co2—N8—C37	−80.48 (9)
N2—Co1—N4—C18	94.90 (10)	N6—Co2—N8—C37	96.98 (9)
C5—N1—C1—C2	−3.7 (2)	C24—N5—C20—C21	−2.2 (2)
Co1—N1—C1—C2	−174.38 (12)	Co2—N5—C20—C21	−171.04 (14)
N1—C1—C2—C3	0.1 (2)	N5—C20—C21—C22	1.6 (3)
C1—C2—C3—C4	2.5 (2)	C20—C21—C22—C23	−0.4 (3)
C2—C3—C4—C5	−1.4 (2)	C21—C22—C23—C24	−0.2 (3)
C1—N1—C5—C4	4.8 (2)	C20—N5—C24—C23	1.6 (2)
Co1—N1—C5—C4	176.75 (12)	Co2—N5—C24—C23	171.75 (14)
C1—N1—C5—C6	−171.34 (13)	C20—N5—C24—C25	−175.09 (14)
Co1—N1—C5—C6	0.57 (15)	Co2—N5—C24—C25	−4.98 (17)
C3—C4—C5—N1	−2.3 (2)	C22—C23—C24—N5	−0.5 (3)
C3—C4—C5—C6	173.47 (15)	C22—C23—C24—C25	175.88 (18)
N1—C5—C6—N2	30.38 (16)	C26—N6—C25—C24	70.86 (15)
C4—C5—C6—N2	−145.69 (15)	C28—N6—C25—C24	−166.58 (12)
C9—N2—C6—C5	−168.43 (11)	Co2—N6—C25—C24	−44.83 (13)
C7—N2—C6—C5	69.04 (14)	N5—C24—C25—N6	33.63 (18)
Co1—N2—C6—C5	−45.60 (12)	C23—C24—C25—N6	−142.96 (17)
C9—N2—C7—C8	124.42 (13)	C28—N6—C26—C27	115.70 (13)
C6—N2—C7—C8	−116.07 (13)	C25—N6—C26—C27	−123.98 (13)
Co1—N2—C7—C8	−3.05 (14)	Co2—N6—C26—C27	−10.28 (14)
Co1—O1—C8—O2	−171.62 (11)	Co2—O5—C27—O6	172.00 (13)
Co1—O1—C8—C7	9.16 (16)	Co2—O5—C27—C26	−8.93 (16)
N2—C7—C8—O2	176.96 (12)	N6—C26—C27—O6	−167.91 (14)
N2—C7—C8—O1	−3.79 (18)	N6—C26—C27—O5	12.98 (18)
C7—N2—C9—C10	−61.72 (16)	C26—N6—C28—C29	−58.19 (16)
C6—N2—C9—C10	176.19 (12)	C25—N6—C28—C29	179.89 (12)
Co1—N2—C9—C10	60.83 (15)	Co2—N6—C28—C29	64.23 (14)
N2—C9—C10—C11	−30.47 (19)	N6—C28—C29—C30	−35.32 (18)
C17—N4—C11—C10	179.54 (13)	C36—N8—C30—C29	177.06 (12)
C18—N4—C11—C10	−58.13 (17)	C37—N8—C30—C29	−61.25 (16)
Co1—N4—C11—C10	64.34 (15)	Co2—N8—C30—C29	61.44 (14)
C9—C10—C11—N4	−35.5 (2)	C28—C29—C30—N8	−30.78 (19)
C16—N3—C12—C13	−3.6 (3)	C35—N7—C31—C32	−3.3 (2)
Co1—N3—C12—C13	−174.26 (14)	Co2—N7—C31—C32	−172.13 (11)
N3—C12—C13—C14	2.0 (3)	N7—C31—C32—C33	0.3 (2)
C12—C13—C14—C15	1.5 (3)	C31—C32—C33—C34	1.6 (2)
C13—C14—C15—C16	−3.4 (3)	C32—C33—C34—C35	−0.6 (2)
C12—N3—C16—C15	1.6 (2)	C31—N7—C35—C34	4.4 (2)
Co1—N3—C16—C15	173.45 (13)	Co2—N7—C35—C34	174.75 (12)
C12—N3—C16—C17	−175.99 (14)	C31—N7—C35—C36	−171.45 (13)
Co1—N3—C16—C17	−4.20 (17)	Co2—N7—C35—C36	−1.10 (15)
C14—C15—C16—N3	1.8 (3)	C33—C34—C35—N7	−2.5 (2)
C14—C15—C16—C17	179.20 (17)	C33—C34—C35—C36	172.89 (14)
C11—N4—C17—C16	−165.84 (13)	N7—C35—C36—N8	31.48 (16)
C18—N4—C17—C16	71.27 (15)	C34—C35—C36—N8	−144.25 (15)
Co1—N4—C17—C16	−44.64 (13)	C30—N8—C36—C35	−168.29 (11)
N3—C16—C17—N4	33.05 (18)	C37—N8—C36—C35	68.88 (14)
C15—C16—C17—N4	−144.50 (16)	Co2—N8—C36—C35	−45.61 (12)
C11—N4—C18—C19	114.02 (14)	C30—N8—C37—C38	125.51 (13)
C17—N4—C18—C19	−125.39 (13)	C36—N8—C37—C38	−114.67 (13)
Co1—N4—C18—C19	−11.69 (14)	Co2—N8—C37—C38	−1.80 (14)
Co1—O3—C19—O4	169.65 (12)	Co2—O7—C38—O8	−169.73 (12)
Co1—O3—C19—C18	−11.81 (16)	Co2—O7—C38—C37	11.39 (16)
N4—C18—C19—O4	−165.58 (13)	N8—C37—C38—O8	174.96 (13)
N4—C18—C19—O3	15.81 (18)	N8—C37—C38—O7	−6.12 (18)
